# METTL3 Promotes the Progression of Gastric Cancer via Targeting the MYC Pathway

**DOI:** 10.3389/fonc.2020.00115

**Published:** 2020-02-26

**Authors:** Dong-Dong Yang, Zhan-Hong Chen, Kai Yu, Jia-Huan Lu, Qi-Nian Wu, Yun Wang, Huai-Qiang Ju, Rui-Hua Xu, Ze-Xian Liu, Zhao-Lei Zeng

**Affiliations:** ^1^State Key Laboratory of Oncology in South China, Department of Medical Oncology of Sun Yat-Sen University Cancer Center, Collaborative Innovation Center for Cancer Medicine, Guangzhou, China; ^2^Shaoguan Municipal Health Bureau, Shaoguan, China; ^3^Department of Medical Oncology and Guangdong Key Laboratory of Liver Disease, The Third Affiliated Hospital of Sun Yat-Sen University, Guangzhou, China

**Keywords:** METTL3, gastric cancer, prognostic factor, MYC target genes, minichromosome maintenance complex component 5, minichromosome maintenance complex component 6

## Abstract

Methyltransferase-like 3 (METTL3), a major component of the N6-methyladenosine (m6A) methyltransferase complex, has been suggested to function as an oncogene in several cancers. However, its biological mechanism and the involved pathways in gastric cancer (GC) remain unknown. Here, we reported that frequent upregulation of METTL3 was responsible for the aberrant m6A levels in gastric carcinoma. On the other hand, a high level of METTL3 was significantly associated with several clinicopathological features and poor survival in patients with GC. The knockdown of METTL3 effectively inhibited cell proliferation and migration and invasion capacity. Moreover, overexpression of METTL3 considerably augmented its oncogenic function. Integrated RNA-seq and m6A-seq analysis first indicated that several component molecules (e.g., MCM5, MCM6, etc.) of MYC target genes were mediated by METTL3 via altered m6A modification. Our work uncovers the oncogenic roles of METTL3 in GC and suggests a critical mechanism of GC progression.

## Introduction

Gastric cancer (GC) is the fifth most common malignancy and is a great burden to patients and health-care systems worldwide. Despite an overall decline in incidence and mortality rates in the past few decades, GC still kills more than 300,000 people per year in China. Although a multidisciplinary treatment (MDT) for GC patients has been developed, trastuzumab has been approved for human epidermal growth factor receptor 2 (HER2)-positive metastatic GC patients, and immunocheckpoint inhibitors have been approved for the third-line treatment of metastatic GC patients, the prognosis of most patients with metastatic GC is far from satisfactory (1–5). Thus, it is necessary to clarify the detailed molecular mechanism underlying GC progression.

N6-methyladenosine (m6A) modification is the most prominent and conservative RNA modification in both prokaryotes and eukaryotes. It is extensively involved in messenger RNA (mRNA) metabolism, including alternative splicing, stability, translation initiation, transportation, and degradation processes. Recent studies have shown that m6A modifications in mRNA play critical roles in cell meiosis, circadian clock control, and cell fate decisions, even in viral infections and immune responses (6–14). M6A modifications are dynamic and reversible processes catalyzed by methyltransferases (writers) and demethylases (erasers). Together with methyltransferase-like 14 (METTL14) and Wilms tumor 1 associated protein (WTAP), methyltransferase-like 3 (METTL3) forms a stable methyltransferase complex that can add a methyl group to RNA. Fat mass and obesity-associated protein (FTO) and alkB homolog 5 (ALKBH5) can function as erasers to deplete m6A modification (15–17). Accumulating evidence has demonstrated that m6A-related enzymes (e.g., METTL3, METTL14, FTO, etc.) can participate in carcinogenesis in several malignancies by mediating m6A modification of target RNAs (18–24). Although several studies have clarified a critical role for METTL3 in GC, the absence of the detailed working mechanism of METTL3 was the most obvious limitation of these studies (25–28). Therefore, in this research, we attempted to investigate the workflow of METTL3-m6A-target RNAs in GC.

In this study, we identified aberrant METTL3 expression and its prognostic value in GC. Then, we confirmed the functional role of METTL3 *in vitro* and *in vivo*. The most interesting thing was the regulation of several key components (e.g., MCM5, MCM6, etc.) of the MYC pathway by METTL3 through mediating m6A modification in their mRNAs. Thus, the findings of our research have provided a fresh view of m6A modification in tumor progression.

## Materials and Methods

### Data Mining From the TCGA Database

The Cancer Genome Atlas (TCGA) mRNA-seq data of 32 normal gastric tissues and 375 primary tumor tissues were downloaded from Firehose (http://gdac.broadinstitute.org/). We calculated the fold change and adjusted *p*-value for all m6A modification-related enzymes via the DESeq2 package, in which an adjusted *p* < 0.05 and fold change >2 was considered to denote a differentially expressed gene.

### Patients and Clinical Databases

Patients enrolled in this study received primary radical or palliative resection without preoperative chemotherapy or radiotherapy at Sun Yat-Sen University Cancer Center (SYSUCC) between January 2007 and February 2013. The patients who met the following criteria were included: (1) pathologically confirmed gastric cancer, (2) patients received radical surgery or palliative surgery, and (3) patients with available clinicopathological information and complete follow-up information. We excluded patients who met the following criteria: (1) patients with synchronous malignant tumors, and (2) patients with incomplete baseline clinicopathological factor information. The median age of all patients was 56 (interquartile range, 50–65). Clinicopathological characteristics including gender, age, pathological tumor–node–metastasis (pTNM) stage, tumor size and grade, invasion depth, neural/vessel invasion, and survival status were described in electronic medical records. All patients were pathologically diagnosed and classified by experienced pathologist according to the 7th edition of the American Joint Committee on Cancer (AJCC) staging system (29). The patients were regularly followed up every 3–6 months until death or dropout, with a median follow-up duration of 41 months [interquartile range (IQR), 21–84 months].

### Cell Culture and Transfection

GES-1, MKN45, MKN74, HGC27, SGC7901, MGC803, and pGCC (primary GC cells) were cultured in Roswell Park Memorial Institute (RPMI)-1640 (Gibco) supplemented with 10% fetal bovine serum (FBS) (Gibco), and 1% antibiotics (penicillin/streptomycin) (Gibco). AGS was maintained in F-12 (Gibco) with 10% FBS and 1% antibiotics. Cells were grown in a 5% CO_2_ incubator at 37°C. Lentiviral vectors expressing non-targeting control RNA (sh#nc and oe#nc), two short hairpin RNAs (shRNAs) (sh#1 and sh#2) targeting METTL3 and oe#METTL3 (overexpression of METTL3) were purchased from Gene Pharma. AGS and SGC7901 cells were incubated with lentivirus and 4 μg/ml polybrene. After 48 h of transfection, 2 μg/ml puromycin was added to the culture medium for the selection of infected cells.

### Cell Functional Assays *in vitro*

Cell proliferation, colony formation, migration, and invasion assays were performed as follows. Briefly, cells were seeded at a density of 1 × 10^3^ cells per well in 96-well plates on day 0. Then, 20 μl [3-(4,5-dimethylthiazol-2-yl)-5- (3-carboxymethoxyphenyl)-2-(4-sulfophenyl)-2H-tetrazolium] (MTS) was added to each well and incubated with cells at 37°C for 2 h on days 1–4. Then, the absorption values at 490 nm were used to measure cell proliferation. For colony formation assays, cells were digested and seeded in six-well plates at 500 cells per well. After 3 weeks of growth, the cells were fixed with methanol and stained with 0.3% crystal violet. Cell migration and invasion were measured using a BioCoat Matrigel Invasion Chamber (Corning) following the manufacturer's instructions.

### *In vivo* Tumor Xenograft

Four to six week-old female BALB/c nude mice (Vital River) were purchased for the construction of subcutaneous tumor xenografts. A total of 2 × 10^6^ GC cells were injected into the flank of nude mice in a 1:1 suspension of BD Matrigel (BD Biosciences) in phosphate-buffered saline (PBS) solution. Vernier calipers were used to detect the formation of xenograft tumors every 4 days. Three weeks after injection, we euthanized nude mice for the measurement of tumor volume and tumor weights.

### RNA m6A Quantification and qRT-PCR

Total RNA from tissues or cell cultures was extracted using TRIzol (Invitrogen) following the manufacturer's protocol. Then, we used an m6A RNA methylation quantification kit (P-9005-48, EpiGentek) to measure the m6A levels in global RNAs. In brief, 200 ng of total RNA from the samples was added to each well and coated at 37°C for 90 min. Then, capture antibody, detection antibody, and enhancer solution were sequentially added to assay wells according to the user guide. We added a developer solution to wells for color development and measured the absorption value at 450 nm. Then, the m6A levels of each well were calculated by standard curve. Quantitative reverse transcription PCR (qRT-PCR) was performed using PrimeScript RT^TM^ Master Mix (RR036Q, TAKARA) and Go Taq® qPCR Master Mix (A6002, Promega). The primers used in this study are listed in Supplementary Table 1.

### Western Blot Analysis

The procedures of Western blot analysis were conducted as described previously (30). The antibodies used for Western blotting in this research were as follows: METTL3 (ab195352, Abcam), MYC (ab32072, Abcam), MCM5 (11703-1-AP, Proteintech), MCM6 (13347-2-AP, Proteintech), and glyceraldehyde 3-phosphate dehydrogenase (GAPDH) (ab181602, Abcam).

### Immunohistochemistry

A dozen tissue microarrays (TMAs) were prepared from 196 paraffin-embedded primary tumor blocks, corresponding adjacent normal mucosa and metastatic lesions. METTL3 immunostaining was performed as previously described (31). The estimation of METTL3 expression was determined by two independent pathologists who were blinded to the clinical data. The staining intensity was scored as 0 (negative), 1 (weak), 2 (moderate), or 3 (strong). The final score of each tissue block was the mean of the products of positive staining rate (0–100%) and intensity score (0–3), ranging from 0 to 300. We applied receiver operating characterizing (ROC) curve analysis to define the optimal cut-off value. The best threshold of METTL3 expression in primary tumors was 145, which meant that patients whose primary tumor score was above that value were grouped into the high expression group (*n* = 69); otherwise, they were grouped into the low expression group (*n* = 127).

### m6A-seq Assays and Data Analysis

Characterization of the cell methylome was carried out by m6A-seq following procedures described previously (30, 32). Briefly, a total of ~100 μg of global cellular RNAs was isolated by a poly(A) mRNA with poly(T) oligo-attached magnetic beads (Invitrogen) (Supplementary Figure 1A). Then, purified poly(A) mRNA samples were fragmented into ~100 nt oligonucleotides under the divalent cations with elevated temperature. Fragmented mRNA was incubated with m6A-specific antibody (No. 202003, Synaptic Systems, Germany) in immunoprecipitation (IP) buffer (50 mM Tris–HCl, 750 mM NaCl, and 0.5% Igepal CA-630) for 2 h at 4°C. The solution was mixed with protein-A beads for m6A immunoprecipitation, and the beads were eluted by elution buffer (1 × IP buffer and 6.7 mM m6A) three times. Both input and m6A IP samples were prepared according to the manufacturer's instructions for sequencing on an Illumina NovaSeq 6000 platform at the LC-BIO Biotech Ltd.

For the data analysis, the enriched m6A peaks were identified by exomePeak for further analysis. The m6A peaks were then annotated by HOMER, and consensus sequence motifs were identified using MEME with the default parameters. Differentially methylated m6A peaks were calculated by limma. The gene expression matrix was quantified and normalized using the RSEM software, and the “DESeq” package was used to find differentially expressed genes. Gene set enrichment analysis (GSEA) was performed to evaluate the pathway differences in expression and m6A modification patterns between the sh#METTL3 and control groups.

### Statistical Analysis

Each cellular experiment was repeated at three biological replicates. The cut-off value of METTL3 was defined using ROC curve analysis. We compared differences of continuous variables by Student's *t*-tests. We compared differences in categorical factors between groups by chi-square test and Fisher's exact test. We compared median values between different groups using the Mann–Whitney test.

In univariate analyses, we estimated the survival difference of distinct variables using the Kaplan–Meier method (log-rank test), and we subsequently identified independent prognostic factors by Cox proportional hazards regression models in multivariate analysis.

All tests were two-tailed, and *P* < 0.05 was considered statistically significant. Statistical analysis was performed using GraphPad Prism 6.0 (GraphPad Software Inc., La Jolla, CA, United States) and R statistical package (R software version 3.4.1; R Foundation for Statistical Computing, Vienna, Austria).

## Result

### Frequent Elevation of METTL3 in GC Was Responsible for the Abnormal m6A Levels in Tumor Tissues

**Figure 1:**
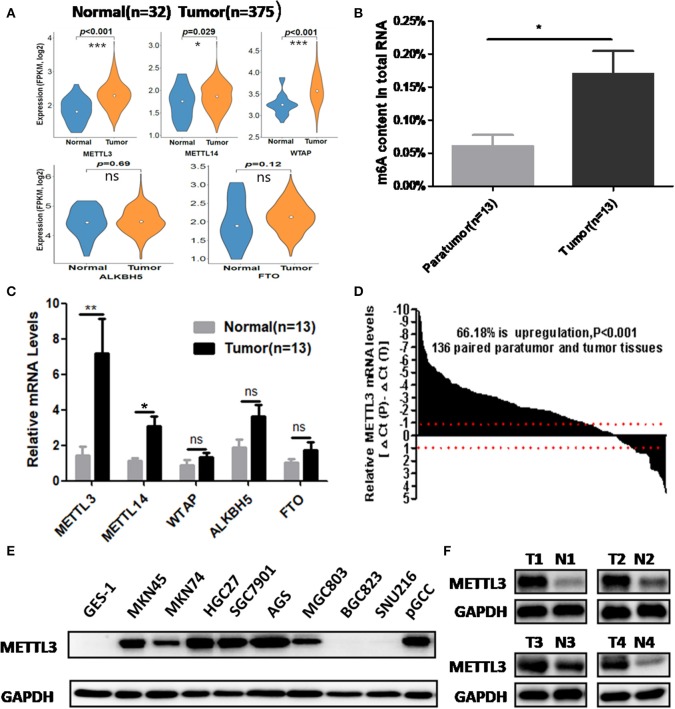
The association between methyltransferase-like 3 (METTL3) upregulation and abnormal m6A levels in gastric cancer (GC). **(A)** Relative expression pf writers and erasers in the The Cancer Genome Atlas (TCGA) database (normal mucosa = 32, tumor tissue = 375). **(B)** Total RNA m6A levels in 13 paired gastric carcinoma tissues and adjacent tissues. **(C)** Relative messenger RNA (mRNA) expression of writers and erasers in the above 13 paired tissues. **(D)** The mRNA expression of METTL3 in 136 paired gastric carcinoma tissues and adjacent tissues. **(E)** METTL3 expression in GES-1, pGCC, and cancer cell lines at the protein level. **(F)** METTL3 protein expression in four paired tumor tissues and adjacent tissues. *p < 0.05, **p < 0.01, ***p < 0.001. ns, non-significant.

We retrieved m6A methyltransferase (METTL3, METTL14, and WTAP) and demethylase (FTO, ALKBH5) expression in GC and normal tissues using online TCGA mRNA-seq data in Firehose. Compared with normal tissues, all three core components of the methyltransferase complex were upregulated, and the obvious alteration of demethylases was not observed in cancerous tissues (Figure 1A). Then, we quantitatively examined m6A levels in the total RNAs of 13 paired cancerous and adjacent tissues. Consistent with the results of the TCGA dataset, we found that m6A levels were increased in the cancerous group compared with their corresponding paratumor group (Figure 1B). Because m6A modification are primarily regulated by the five enzymes above, we explored the mRNA levels of these genes in the corresponding 13 paired tissues. As shown in Figure 1C, the mRNA expression of METTL3 and METTL14 was significantly elevated in GC tissues. In contrast, the expression of WTAP, FTO, or ALKBH5 was not significantly altered in tumor tissues. Next, we further estimated METTL3 and METTL14 expression in a larger cohort containing 136 paired tumor and paratumor tissues. In this sample cohort, METTL3 expression was markedly elevated in 66.18% (90/136) of patients with GC (Figure 1D). However, elevation of METTL14 was detected in 44.11% (60/136) of patients (Supplementary Figure 1B). Considering the higher percentage of METTL3 elevation in GC and its catalytic roles in the methyltransferase complex, we selected METTL3 as the candidate molecule for aberrant m6A modification in GC.

Simultaneously, we determined METTL3 expression in nine GC cell lines and GES-1 (an immortalized gastric epithelial cell line) by Western blot analysis. The results indicated that METTL3 protein levels in most cancer cell lines (except for BGC823 and SUN216) were higher than those in GES-1 (Figure 1E). In addition, METTL3 expression was also elevated in four cases of gastric carcinoma tissues (Figure 1F). Based on the above evidence, we deduced that the upregulation of METTL3, a leading cause of higher m6A levels in GC, occurred frequently in patients who suffered from this deadly disease.

### Upregulation of METTL3 Was Associated With Poor Patient Survival

**Figure 2:**
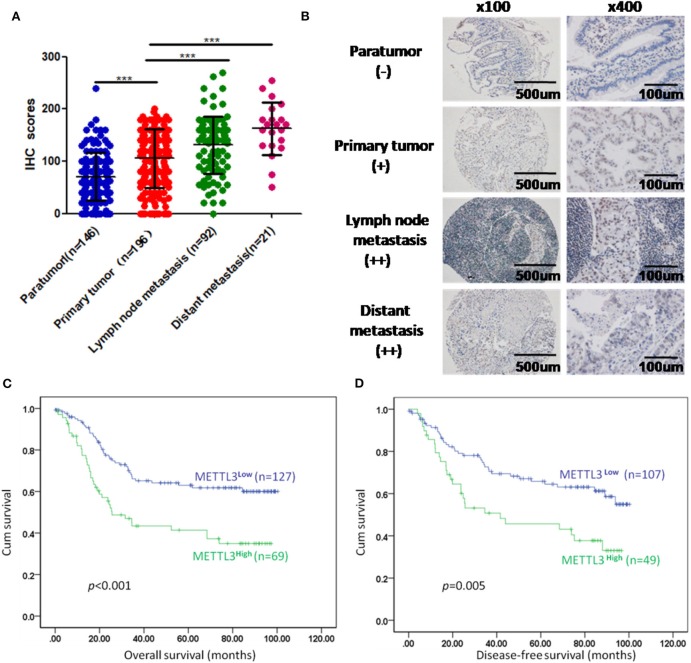
Immunohistochemistry (IHC) scores of methyltransferase-like 3 (METTL3) in different types of tissues and Kaplan–Meier survival curves in gastric cancer (GC) patients. **(A)** IHC scores of METTL3 in four different types of tissues from 196 patients with GC (paratumor, mean ± SD = 70.74 ± 46.29; primary tumor, mean ± SD = 105.60 ± 56.16; lymph node metastasis, mean ± SD = 130.80 ± 55.31; distant metastasis: mean ± SD = 162.6 ± 50.19). **(B)** Immunohistochemical staining of METTL3 in four different types of tissues from the same patient. **(C)** Overall survival analysis in all GC patients (*n* = 196). **(D)** Disease-free survival analysis in GC patients who were first diagnosed with tumor–node–metastasis (TNM) stage I, II, or III (*n* = 156). ***p < 0.001.

We enrolled 196 patients diagnosed with GC in our hospital to determine the prognostic value of METTL3 in these patients. In total, we examined METTL3 expression in 455 paraffin-embedded blocks (paratumor, *n* = 146; primary tumor, *n* = 196; lymph node metastasis, *n* = 92; distant metastasis, *n* = 21). The immunohistochemistry (IHC) scores of METTL3 in the primary tumor group were significantly higher than those in the paratumor group (Figure 2A, *p* < 0.001). Meanwhile, METTL3 expression in lymph node metastatic and distant metastatic tissues is significantly higher than that of primary tumor tissues (Figure 2A). Representative IHC images of different tissues are shown in Figure 2B. Furthermore, matched normal and cancerous tissues from this cohort also showed similar differences (Supplementary Figures 1C,D).

Subsequently, we further assessed the relationship between METTL3 expression and other clinicopathological variables. As shown in Table 1, METTL3 expression was significantly associated with tumor size (*p* = 0.011) and distant metastasis (*p* = 0.040) and TNM stage (*p* = 0.035). Other remaining factors had no statistical correlation with METTL3 (Table 1). Moreover, METTL3 expression in the primary tumor was elevated with increased M stage and TNM stage (Supplementary Figures 1E,F). Then, we performed Kaplan–Meier survival curve analysis (log-rank test) to confirm the prognostic value of METTL3 expression in GC. As shown in Figures 2C,D, GC patients with higher METTL3 expression had significantly shorter overall survival (OS) (*p* < 0.001) and disease-free survival (DFS) (*p* = 0.005). In addition, in the tumor size ≤5 cm group and grade III group, high METTL3 expression was associated with worse OS and DFS (Supplementary Figure 2).

**Table 1:** The relationship between METTL3 expression and clinicopathological parameters.

**Variable**	**No. of patients (%)**	**METTL3 low expression, n (%)**	**METTL3 high expression, n (%)**	***P*-value**
Total	196 (100%)	127 (64.8)	69 (35.2)	
Gender				0.153
Female	65 (33.2)	47 (72.3)	18 (27.7)	
Male	131 (66.8)	80 (61.1)	51 (38.9)	
Age				0.881
≤55	89 (45.4)	57 (64.0)	32 (36.0)	
>55	107 (54.6)	70 (65.4)	37 (34.6)	
T stage				0.505
T_1−2_	25 (12.8)	18 (72.0)	7 (28.0)	
T_3−4_	171 (87.2)	109 (63.7)	62 (36.3)	
N stage				0.249
N_0_	56 (28.6)	40 (71.4)	16 (28.6)	
N_1−3_	140 (71.4)	87 (62.1)	53 (37.9)	
M stage				0.040
M0	156 (79.6)	107 (68.6)	49 (31.4)	
M1	40 (20.4)	20 (50.0)	20 (50.0)	
Tumor size				0.011
≤5	129 (65.8)	92 (71.3)	37 (28.7)	
>5	67 (34.2)	35 (52.2)	32 (47.8)	
Grade				0.069
I–II	55 (28.1)	30 (54.5)	25 (45.5)	
III	141 (71.9)	97 (68.8)	44 (31.2)	
Invasion depth				0.396
Not whole layer	28 (14.3)	16 (57.1)	12 (42.9)	
Whole layer	168 (85.7)	111 (66.1)	57 (33.9)	
Neural invasion				0.176
No	52 (26.5)	38 (73.1)	14 (26.9)	
Yes	144 (73.5)	89 (61.8)	55 (38.2)	
Vessel invasion				0.356
No	75 (38.3)	52 (69.3)	23 (30.7)	
Yes	121 (61.7)	75 (62.0)	46 (38.0)	
TNM stage				0.035
I–II	109 (55.6)	78 (71.6)	31 (28.4)	
III–IV	87 (44.4)	49 (56.3)	38 (43.7)	
Status				0.001
Alive	113 (57.7)	84 (74.3)	29 (25.7)	
Dead	83 (42.3)	43 (51.8)	40 (48.2)	

Importantly, the results of univariate analysis revealed that well-recognized clinical prognostic parameters and METTL3 expression were significantly correlated with OS and DFS (Table 2). Multivariate analysis confirmed that METTL3 expression was an independent prognostic factor for OS [hazard ratio (HR), 1.741; 95% confidence interval (CI), 1.123–2.698; *p* = 0.013] and DFS (HR, 1.936; 95% CI, 1.189–3.512; *p* = 0.008). Collectively, these findings suggested that high METTL3 expression was an independent prognostic factor of poor overall survival in GC patients.

**Table 2:** Univariate and multivariate analysis of OS in 196 patients (TNM stage I–IV) and DFS in 156 patients (TNM stage I–III).

**Variables**	**OS**				**DFS**			
	**Univariate**		**Multivariate**		**Univariate**		**Multivariate**	
	**HR (95% CI)**	***P*-value**	**HR (95% CI)**	***P*-value**	**HR (95% CI)**	***P*-value**	**HR (95% CI)**	***P*-value**
Gender	1.104 (0.694–1.756)	0.676			1.636 (0.921–2.907)	0.093		
Male vs. Female								
Age	0.842 (0.547–1.295)	0.433			0.846 (0.523–1.224)	0.495		
>55 vs. ≤55								
Tumor size	1.642 (1.064–2.532)	0.025*			1.652 (1.016–2.688)	0.043*		
>5 cm vs. ≤5 cm								
Grade	0.733 (0.459–1.172)	0.733			1.726 (0.881–3.382)	0.111		
III vs. I–II								
Invasion depth	2.851 (1.242–6.574)	0.014*			2.446 (1.057–5.658)	0.037*		
Whole layer vs. Not whole layer								
Neural invasion	2.153 (1.211–3.892)	0.009*			1.956 (1.085–3.528)	0.026*		
Yes vs. No								
Vessel invasion	2.456 (1.470–4.102)	0.001*			1.904 (1.136–3.192)	0.015*		
Yes vs. No								
T stage	3.781 (1.384–10.330)	0.009*			3.429 (1.248–9.422)	0.005*		
T_3−4_ vs. T_1−2_								
N stage	4.060 (2.092–7.875)	<0.001*	2.175 (1.046–4.521)	0.037	2.931 (1.568–5.480)	0.001*	2.029 (1.018–4.046)	0.044
N_1−3_ vs. N_0_								
M stage	4.965 (3.098–7.981)	<0.001*	2.321 (1.361–3.959)	0.002				
M_1_ vs. M_0_								
TNM stage	4.730 (2.949–7.587)	<0.001*	2.408 (1.337–4.334)	0.003	2.692 (1.635–4.384)	<0.001*	1.727 (1.003–2.973)	0.045
III–IV vs. I–II								
METTL3	2.177 (1.414–3.353)	<0.001*	1.741 (1.123–2.698)	0.013	1.991 (1.226–3.324)	0.001*	1.936 (1.189–3.512)	0.008
High vs. Low expression								

HR, hazard ratio; CI, confidence interval; TNM, tumor-node-metastasis. *p < 0.05.

### Knockdown of METTL3 Inhibited the Growth and Metastasis of GC

**Figure 3:**
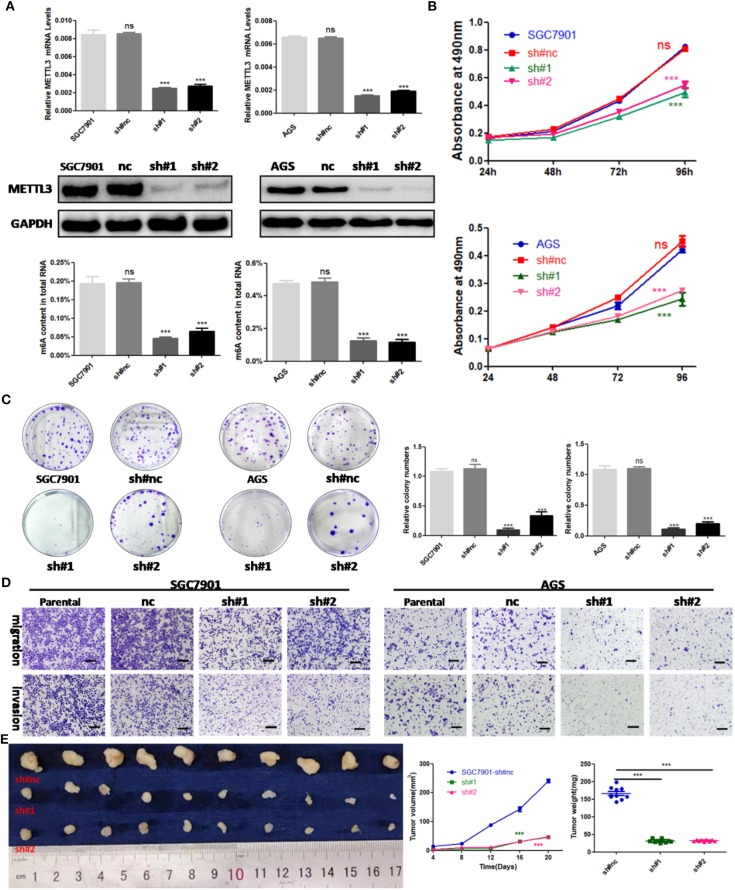
The effects of methyltransferase-like 3 (METTL3) knockdown on cell function *in vitro* and *in vivo*. **(A)** METTL3 knockdown efficiency and m6A levels of total RNAs in SGC7901 and AGS. **(B)** Cell proliferation assays after METTL3 knockdown in SGC7901 and AGS cells. **(C)** Colony formation assays of METTL3 knockdown in SGC7901 and AGS cells. **(D)** Transwell migration and invasion assays after METTL3 knockdown in SGC7901 and AGS. **(E)** The nude mice xenograft models (*N* = 5) of METTL3 knockdown SGC7901 cells. ***p < 0.001. ns, non-significant. All experiments were repeated three times. Scale bars = 50 μm.

To explore the functional roles of METTL3 in GC, we stably knocked down METTL3 in two GC cell lines, SGC7901 and AGS, using two independent shRNAs (sh#1, sh#2). Then, qRT-PCR and Western blotting were conducted to confirm the high knocking efficiency of METTL3. As expected, once successfully knocking down METTL3, m6A levels in SGC7901 and AGS sharply reduced (Figure 3A). We performed *in vitro* functional assays to determine the role of METTL3 in the proliferation, colony formation, migration, and invasion of GC cells. The results of the MTS assays and colony-forming assays indicated that depletion of METTL3 could obviously suppress proliferation and colony-forming abilities of GC cells (Figures 3B,C). In the migration and invasion assays, the silencing of METTL3 significantly inhibited the migratory and invasive abilities of SGC7901 and AGS cells (Figure 3D).

SGC7901 infected with sh#METTL3 and control lentiviruses were used to establish a subcutaneous tumor xenograft in nude mice. We observed xenograft growth for 3 weeks and found that METTL3 silencing could suppress tumor growth *in vivo* (Figure 3E). All in all, these findings *in vitro* and *in vivo* revealed the oncogenic roles of METTL3 in GC.

### METTL3 Overexpression Enhances Migration and Invasion *in vitro* and Promotes Proliferation *in vivo*

**Figure 4:**
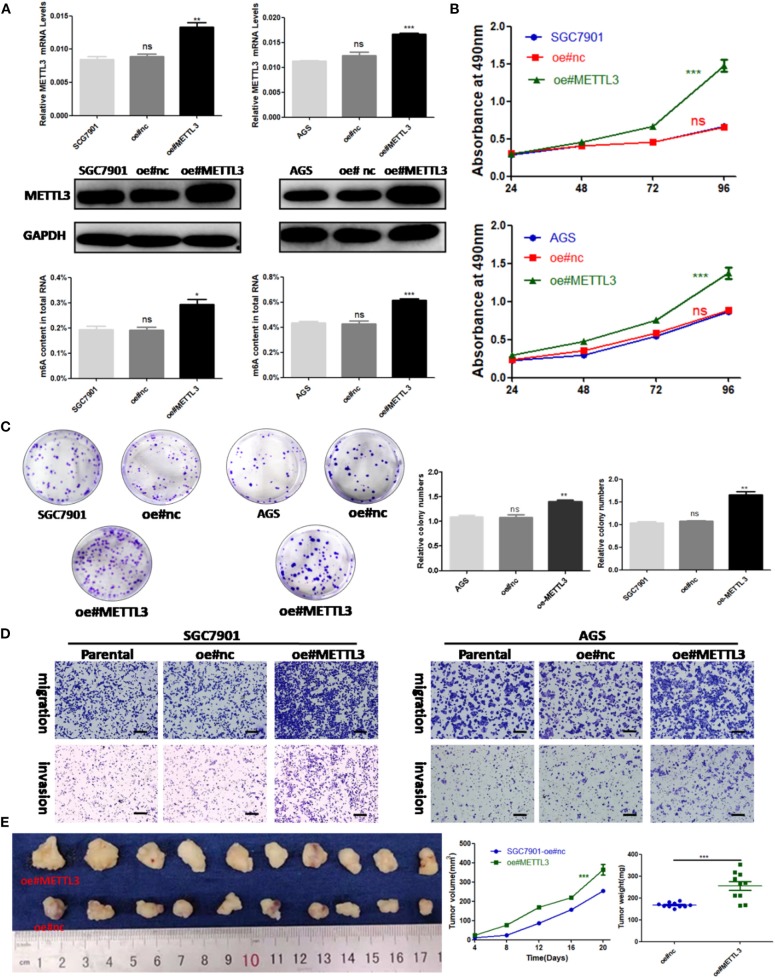
Methyltransferase-like 3 (METTL3) overexpression enhances migration and invasion *in vitro* and promotes proliferation *in vivo*. **(A)** METTL3 overexpression efficiency and m6A levels of total RNA in SGC7901 and AGS. **(B)** Cell proliferation assays of METTL3-overexpressing SGC7901 and AGS cells. **(C)** Colony formation assays of METTL3 overexpressing SGC7901 and AGS cells. **(D)** Transwell migration and invasion assays of METTL3-overexpressing SGC7901 and AGS cells. **(E)** The nude mice xenograft models (*N* = 5) of METTL3 overexpressing SGC7901 cells. *p < 0.05, **p < 0.01, ***p < 0.001. ns, non-significant. All experiments were repeated three times. Scale bars = 50 μm.

Considering the findings of the functional assays above, we were motivated to determine if overexpression of METTL3 could promote GC progression. Then, control lentivirus and lentivirus for overexpressing endogenous METTL3 were used to transfect SGC7901 and AGS cells. The upregulation of METTL3 was verified by qRT-PCR and Western blot analysis. In addition, we observed elevated m6A levels in cells overexpressing METTL3 (Figure 4A). Similarly, cell growth and colony-forming abilities were increased upon METTL3 overexpression as well as migratory and invasive capacities (Figures 4B–D). In nude mouse xenograft models, tumors induced by METTL3-overexpression cells showed significantly larger tumor volumes and heavier tumor weights than those in the control group (Figure 4E). Taken together, METTL3 may act as an oncogene in GC by potentiating cellular proliferation and metastasis.

### METTL3-Dependent m6A Modification Regulated the MYC Pathway

**Figure 5:**
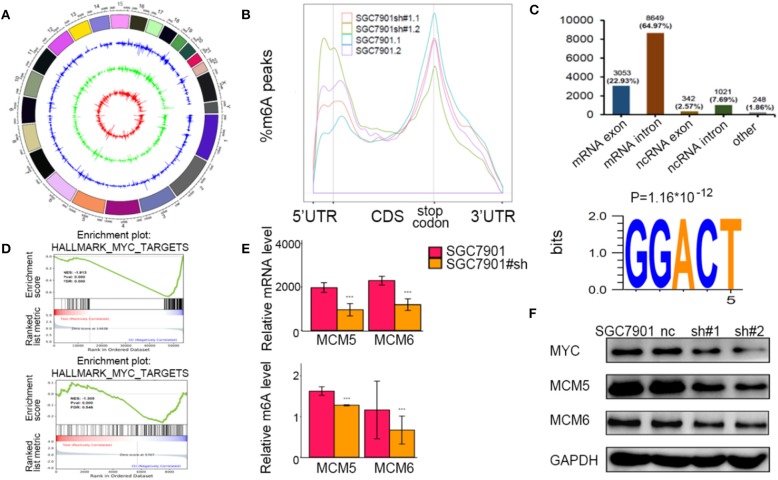
Identification of the potential m6A-dependent targets of methyltransferase-like 3 (METTL3) in GC. **(A)** Identification of m6A peaks by two algorithms. The layers from outer to inner represent the m6A peaks identified by exomePeak, masc2, and the overlap from both algorithms. **(B)** The density distribution of m6A peaks in messenger RNA (mRNA) transcripts. **(C)** Distribution of the m6A peaks in exonic or intronic regions of protein-coding and non-coding genes and in other regions. The consensus motif from all identified m6A peaks. **(D)** Gene set enrichment analysis determined by both RNA-seq and m6A-seq selected MYC pathway as downstream of METTL3. **(E)** Relative m6A levels of MCM5 and MCM6 upon depletion of METTL3. **(F)** The protein levels of MCM5, MCM6, and MYC upon depletion of METTL3. *p < 0.05, **p < 0.01, ***p < 0.001. ns, non-significant.

To identify the potential targets methylated by METTL3 in GC, we performed RNA-seq and m6A-seq in SGC7901 cell lines. Then, we applied two algorithms widely used in m6A-seq analysis (masc2 and exomePeak) to confirm the profiles of m6A peaks (Figure 5A). In line with previously published works, we identified 24,195 peaks in 13,322 genes. The distribution of the identified m6A peaks was the highest enriched near the stop codon, with a higher percentage in the mRNA intron and mRNA exon (Figures 5B,C). Consistent with other cell types, the consensus motif of all m6A sites was GGACT (Figure 5C).

To explore the METTL3-m6A-target genes, we performed GSEA using the hypo-methylation site lists combined with differentially expressed genes upon METTL3 depletion. There were multiple signaling pathways that were positively/negatively associated with METTL3 depletion in this merged analysis (Supplementary Table 2). MYC target genes had a significant decrease at both the m6A and mRNA levels after METTL3 knockdown (Figure 5D). Here, we showed decreases in MCM5 and MCM6 (key components of MYC target genes) at epigenetic levels (Figure 5E). In addition, the protein levels of MYC, MCM5, and MCM6 were decreased with METTL3 knockdown in SGC7901 cells (Figure 5F). These findings strongly indicated that MYC target genes were downstream of METTL3 in GC.

## Discussion

In the past few years, METTL3, with other writers and erasers, has been reported to extensively take part in tumorigenesis (33–35). It was reported that METTL3 could participate in glioblastoma tumorigenesis by enhancing glioma stem-like cell (GSC) maintenance and radioresistance. In contrast, another study showed that METTL3 could inhibit GSC growth and self-renewal by decreasing the expression of a series of oncogenes (ADAM19, EPHA3, and KLF4). Several studies have identified that METTL3 can promote cancer cell proliferation and invasion by upregulating downstream key oncogenes via post-transcriptional modification. However, the results from an American research team indicated that METTL3 can increase the invasiveness of lung cancer cells by initiating the translation of oncogenes (EGFR, TAZ, and MAPKAPK2) (36–40).

The roles METTL3 have been identified in several studies of GC. Zhang et al. showed that reduced m6A levels could activate Wnt/phosphatidylinositol-3-kinase (PI3K)-protein kinase B (AKT) signaling and promote malignant phenotypes in GC cells. Lin and collaborators found that METTL3 could promote the proliferation and mobility of GC cells by activating the AKT pathway. The results of Liu and coworkers indicated that METTL3 knockdown reduces α-smooth muscle actin and that METTL3 is an adverse factor in patients with GC (25–27). However, these studies explored the METTL3 regulatory network in GC simply by bioinformatics analysis. In Yue's research, the results of m6A-seq indicated that METTL3 could enhance ZMYM1 mRNA stability, which facilitates the EMT program and metastasis of GC (41). Similarly, in our research, we explored the downstream targets of METTL3 by high-throughput m6A-seq and in-depth bioinformatics analysis in GC cell lines and comprehensive experiments. Specifically, we first observed that frequent upregulation of METTL3 rather than METTL14 or WTAP was responsible for elevated m6A levels and poor survival prognosis. Then, we confirmed the carcinogenesis of METTL3 by silencing or overexpressing METTL3 in cells. The novelty of this study was the identification of MYC and its target genes downstream of METTL3.

The MYC oncogene and its target genes contribute to the genesis of many human cancers. The upstream and downstream of MYC were extensively involved in cell growth and proliferation as well as nutrient metabolism. Indeed, antitumor strategies targeting MYC have offered new cancer therapeutic opportunities (42). Several studies have indicated that METTL3, METTL14, or FTO could affect the stability of MYC mRNA via the alteration of m6A abundance (39, 43, 44). However, in the current research, we did not observe influences on the relative m6A abundance of MYC transcripts by METTL3 depletion. Indeed, MYC and its downstream proteins were degraded upon METTL3 depletion. The precise mechanism of how METTL3 manipulates MYC expression remains unknown in GC and needs to be further explored.

More importantly, we identified the key components of the MYC pathway as direct target genes of METTL3 in GC. MCM5 and MCM6, which belong to the minichromosome maintenance (MCM) family, assembled into the prereplication complex for the initiation of eukaryotic genome replication. It was reported that high MCM5 expression was associated with poor clinicopathological parameters and poor survival in GC. According to a previous report, MCM6 can serve as a prognostic predictor and promote metastasis in hepatocellular carcinoma (HCC) (45–47). In our research, m6A abundances of MCM5 and MCM6 transcripts were sharply decreased upon the depletion of METTL3 in cells. Some published studies have shown that certain gene transcripts are degraded upon the downregulation of m6A modification in several cancers. Thus, we presumed that the possible regulatory mechanisms were a decrease in the stability of MCM5 and MCM6 transcripts mediated by METTL3 depletion. The exact mechanism requires further experiments.

With the continuous improvement of m6A-seq and other related detection technologies, we could further detect the precise modality of METTL3 in cancers (48). Currently, three FTO inhibitors (Rhein, MA2, and R-2HG) have effective anticancer potential (34, 36, 49). Future efforts could be made to develop inhibitors targeting METTL3.

In conclusion, our study highlights a new pathway of METTL3-mediated m6A modification in the epigenetic silencing of MYC downstream molecules in GC, which can provide a new strategy for more careful surveillance and aggressive therapeutic intervention.

## Data Availability Statement

The RNA-seq and m6A-seq data from METTL3 knockdown or non-treated SGC7901 have been deposited in BioProject under accession PRJNA595769. Other data generated during this study are not publicly available, and we are willing to share it upon reasonable request.

## Ethics Statement

The studies involving human participants were reviewed and approved by Institute Research Ethics Committee of Cancer Center of Sun Yat-sen University. The patients/participants provided their written informed consent to participate in this study. The animal study was reviewed and approved by Institute Research Ethics Committee of Cancer Center of Sun Yat-sen University.

## Author Contributions

D-DY, Z-HC, and Z-LZ designed the study, acquired the data, and wrote the manuscript. D-DY, Q-NW, YW, Z-LZ, and J-HL collected cell samples from the Pathology Department and did the experiments *in vitro* and *in vivo*. KY and Z-XL interpreted and analyzed the data. R-HX, H-QJ, and Z-LZ revised and approved the final version of the manuscript.

### Conflict of Interest

The authors declare that the research was conducted in the absence of any commercial or financial relationships that could be construed as a potential conflict of interest.
