# Supplementary material for: METTL3 Promotes the Progression of Gastric Cancer via Targeting the MYC Pathway
Source: Front Oncol. 2020 Feb 26;10:115. doi: 10.3389/fonc.2020.00115 (PMC7054453; doi:10.3389/fonc.2020.00115)
Supplement: Supplementary file 1 [file Data_Sheet_1.pdf]

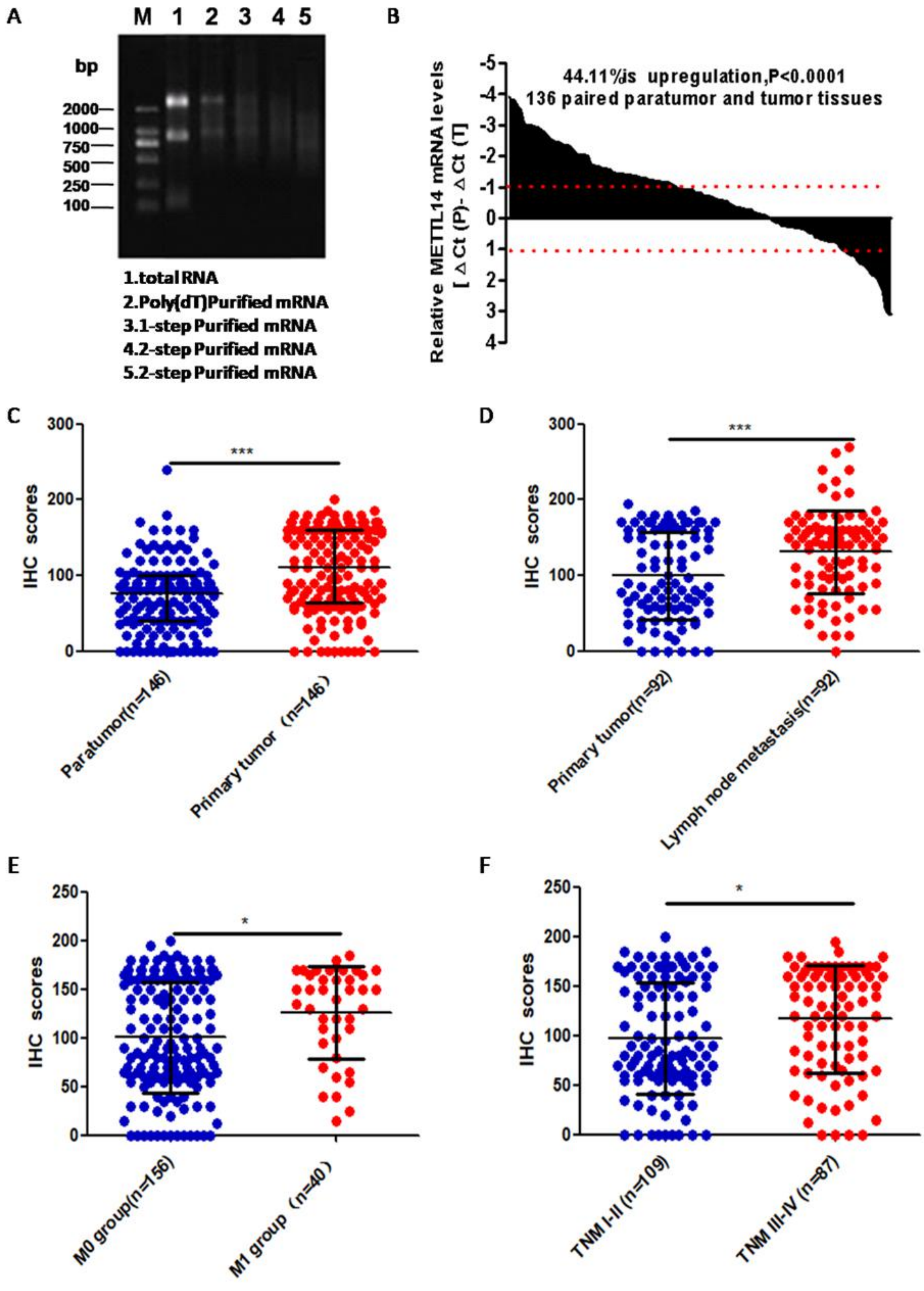

Supplementary Figure 1. (A) Identification of purified mRNA. (B) The mRNA expression of METTL14 in 136 paired gastric carcinoma tissues and adjacent tissues.(C) IHC scores of 146

paired paratumor and primary tumor tissues (paratumor: mean±SD=70.74±46.29; primary tumor: mean±SD=106.50±55.21). (D) IHC scores of 92 paired primary tumor and lymph node metastasis tissues (primary tumor: mean ± SD=99.35 ± 57.68; lymph node metastasis: mean±SD=130.80±55.31) (E) IHC scores of primary tumor tissue from different M stage subsets (M_0 stage: mean±SD=100.20±57.40, M_1 stage: mean±SD=127.40±46.75). (F) IHC scores of primary tumor tissue from different TNM stage subsets. (TNM stage Ⅰ-Ⅱ: mean±SD=96.95±56.40; TNM stage Ⅲ-Ⅳ: mean±SD=116.50±54.11). ***: $p<0.001$, *: $p<0.05$.

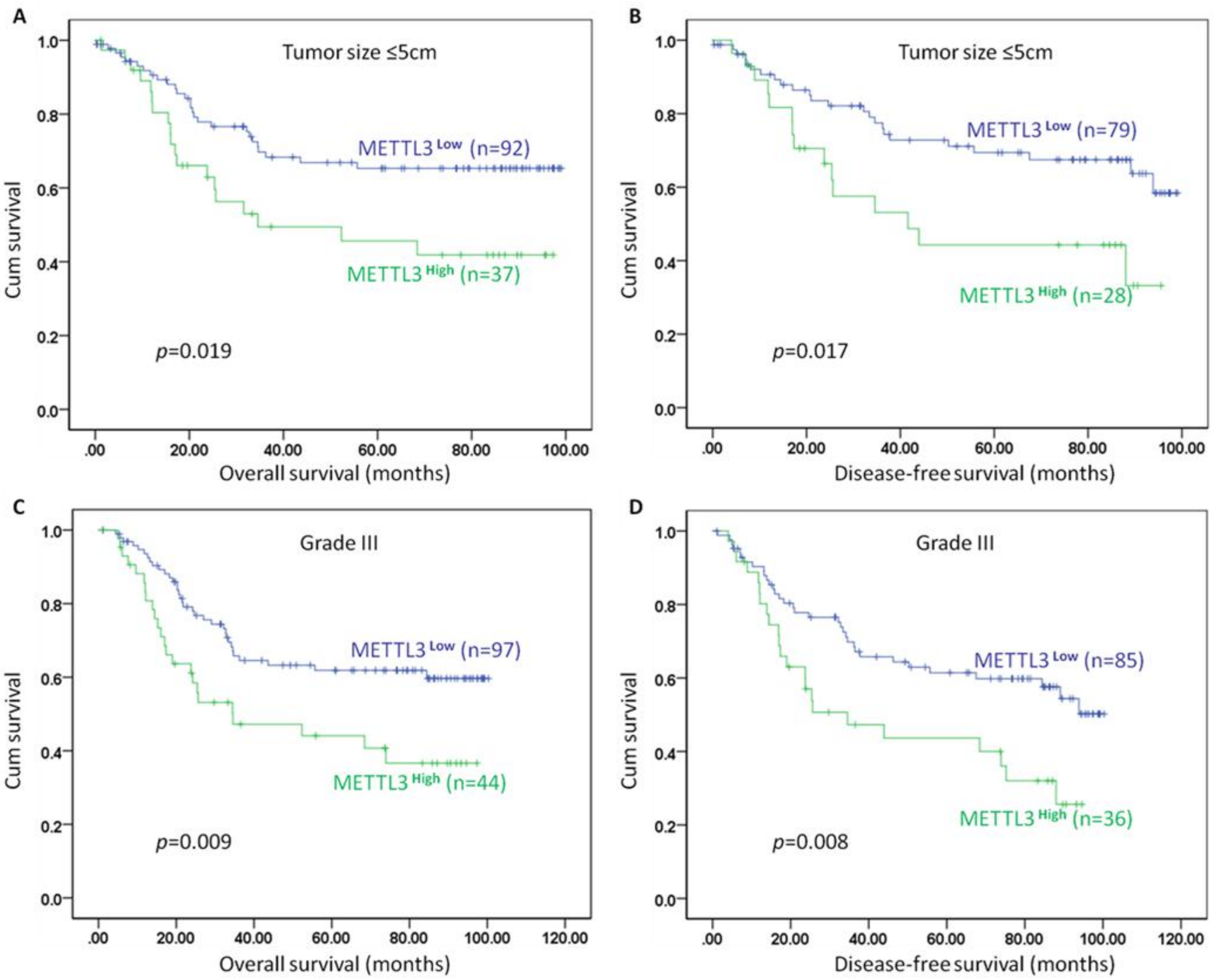

Supplementary Figure 2. Kaplan-Meier curves of overall survival and disease-free survival were stratified by METTL3 expression in different subgroups. (A and B) Tumor size ≤5 cm subgroup; the DFS analysis in this subgroup enrolled GC patients who were first diagnosed with TNM stage Ⅰ, Ⅱ or Ⅲ (n=107). (C and D) Pathological grade Ⅲ subgroup; the DFS analysis in this subgroup enrolled GC patients who were first diagnosed with TNM stage Ⅰ, Ⅱ or Ⅲ (n=121).

Supplementary table 1: The sequences of primers used in this research.

Primers	Sequences (5’ to 3’)
METTL3-Forward Primer	TTGTCTCCAACCTTCCGTAG
METTL3-Reverse Primer	CCAGATCAGAGAGGTGGTGTAG
METTL14-Forward Primer	AGTGCCGACAGCATTGGTG
METTL14-Reverse Primer	GGAGCAGAGGTATCATAGGAAGC
WTAP-Forward Primer	CTTCCCAAGAAGGTTCGATTGA
WTAP-Reverse Primer	TCAGACTCTCTTAGGCCAGTTAC
FTO-Forward Primer	ACTTGGCTCCCTTATCTGACC
FTO-Reverse Primer	TGTGCAGTGTGAGAAAGGCTT
ALKBH5-Forward Primer	CCCGAGGGCTTCGTCAACA
ALKBH5-Reverse Primer	CGACACCCGAATAGGCTTGA
GAPDH-Forward Primer	GAGAAGGCTGGGGCTCATTTGCA
GAPDH-Reverse Primer	TTGGCCAGGGGTGCTAAGCAGT

Supplementary table 2: The pathways affected by METTL3 knockdown in GC at the mRNA and m6A levels.

Pathways downregulated after METTL3 knockdown at mRNA level			
NAME	SIZE	NES	NOM p-value
MYC_TARGETS_V1	191	-1.9131314	0
OXIDATIVE_PHOSPHORYLATION	192	-1.8207923	0
E2F_TARGETS	185	-1.619965	0
MYC_TARGETS_V2	55	-1.4818718	0.00877193
MTORC1_SIGNALING	186	-1.2860876	0
G2M_CHECKPOINT	184	-1.1694806	0.09090909
DNA_REPAIR	134	-1.1257797	0.21428572
CHOLESTEROL_HOMEOSTASIS	65	-0.90599036	0.6883117
Pathways upregulated after METTL3 knockdown at mRNA level			
NAME	SIZE	NES	NOM p-value
TNFA_SIGNALING_VIA_NFKB	184	1.6082172	0
INTERFERON_ALPHA_RESPONSE	90	1.5658641	0
INTERFERON_GAMMA_RESPONSE	187	1.538817	0
KRAS_SIGNALING_UP	180	1.3326728	0.001007049
APOPTOSIS	148	1.35566	0.002053388
INFLAMMATORY_RESPONSE	189	1.3254582	0.003045685
COMPLEMENT	183	1.2742603	0.009146341
IL6_JAK_STAT3_SIGNALING	79	1.3470669	0.017039403
COAGULATION	130	1.280277	0.017708333
UV_RESPONSE_DN	128	1.2808105	0.018614272

P53_PATHWAY	184	1.2369596	0.022289768
EPITHELIAL_MESENCHYMAL_TRANSITION	182	1.2421782	0.031408306
HYPOXIA	189	1.211722	0.047667343
Pathways downregulated after METTL3 knockdown at m6A level			
NAME	SIZE	NES	NOM p-value
OXIDATIVE_PHOSPHORYLATION	164	-2.1449418	0
MYC_TARGETS_V1	172	-1.3048757	0
XENOBIOTIC_METABOLISM	112	-1.2542256	0.022727273
GLYCOLYSIS	149	-1.1010782	0.2173913
APICAL_SURFACE	21	-1.063103	0.36614174
ALLOGRAFT_REJECTION	87	-0.9793832	0.5
FATTY_ACID_METABOLISM	108	-0.9763336	0.6039604
HYPOXIA	130	-0.9683384	0.56790125
DNA_REPAIR	116	-0.96514165	0.4950495
UNFOLDED_PROTEIN_RESPONSE	88	-0.9263118	0.6
SPERMATOGENESIS	59	-0.92570823	0.5945946
REACTIVE_OXIGEN_SPECIES_PATHWAY	34	-0.9131306	0.5963303
MTORC1_SIGNALING	163	-0.8500072	0.91525424
ADIPOGENESIS	135	-0.8096232	0.9868421
TGF_BETA_SIGNALING	35	-0.73800194	0.8995215
Pathways upregulated after METTL3 knockdown at m6A level			
NAME	SIZE	NES	NOM p-value
IL6_JAK_STAT3_SIGNALING	42	1.4609499	0.023690773
